# Tuberculosis in people of Ukrainian origin in the European Union and the European Economic Area, 2019 to 2022

**DOI:** 10.2807/1560-7917.ES.2024.29.12.2400094

**Published:** 2024-03-21

**Authors:** Krista Stoycheva, Veronica Cristea, Csaba Ködmön, Senia Rosales-Klintz, Dominik Zenner, Anca Vasiliu, Marieke van der Werf, Christoph Lange

**Affiliations:** 1 Division of Clinical Infectious Diseases, Research Center Borstel, Borstel, Germany; 2 Respiratory Medicine & International Health, University of Lübeck, Lübeck, Germany; 3 European Centre for Disease Prevention and Control (ECDC), Stockholm, Sweden; 4 Wolfson Institute of Population Health, Barts and the London School of Medicine and Dentistry, Queen Mary University of London, London, United Kingdom; 5 German Center for Infection Research (DZIF), TTU-TB, Hamburg-Lübeck-Borstel-Riems, Germany; 6 Baylor College of Medicine and Texas Children’s Hospital, Houston, USA

**Keywords:** tuberculosis, TB, Ukraine, Russia, war, migration, conflict, EU/EEA, COVID-19, DR, drug resistance, RR/MDR, pre-XDR, XDR, Europe

## Abstract

Approximately five million Ukrainians were displaced to the EU/EEA following the Russian invasion of Ukraine. While tuberculosis (TB) notification rates per 100,000 Ukrainians in the EU/EEA remained stable, the number of notified TB cases in Ukrainians increased almost fourfold (mean 2019–2021: 201; 2022: 780). In 2022, 71% cases were notified in three countries, and almost 20% of drug-resistant TB cases were of Ukrainian origin. Targeted healthcare services for Ukrainians are vital for early diagnosis and treatment, and preventing transmission.

Ukraine is one of the countries in the World Health Organization (WHO) European Region with the highest number of notified cases of tuberculosis (TB) [1-4]. In 2021, the TB notification rate in Ukraine (45.5 cases/100,000 population) was six times higher than the European Union and European Economic Area (EU/EEA) average rate for the same year (7.4/100,000) [4]. Since February 2022, approximately 5 million individuals of Ukrainian origin have fled to EU/EEA countries [5]. We assessed the impact of the arrival of Ukrainians (defined as individuals holding Ukrainian citizenship or born in Ukraine) on the TB epidemiology in the EU/EEA to inform strategies for TB prevention, control and treatment.

## Tuberculosis notifications among Ukrainians in the EU/EEA

We analysed the TB notifications in Ukrainians in the EU/EEA between 2019 and 2022 using TB surveillance data from The European Surveillance System (TESSy), hosted by the European Centre for Disease Prevention and Control (ECDC). Data for 2023 were not available through TESSy at the time of the analysis. For Latvia, data for 2019 and 2020 were obtained directly from the Centre for Disease Prevention and Control of the Republic of Latvia and for Liechtenstein no 2019 data were reported to TESSy. The dataset included the total number of notified cases per year. TB cases were recorded according to the EU case definitions [6]. This included new and relapse cases, as well as re-registered TB cases (previously treated cases not meeting the relapse criteria) [2]. Case origin was assigned either by country of birth or citizenship, varying with the preference of the reporting country [4].

The country-specific and total EU/EEA TB notification rates were calculated using published figures from the Statistical Office of the European Union (Eurostat) on Ukrainian citizens with usual residence in the EU/EEA on 1 January of each year or, where unavailable, on residence permit holders as of 31 December the previous year [7]. The 2021 population dataset for Estonia was retrieved through Statistics Estonia [8]. For 2022, these datasets were combined with the number of approved applications of Ukrainians for temporary protection as of 31 December 2022 [7]. The temporary protection dataset only provides information by citizenship. Therefore, to improve comparability between the time points, we used citizenship in all population datasets. Rates by country of birth (where available) are provided in Supplementary Figure S1.

**Figure 1:**
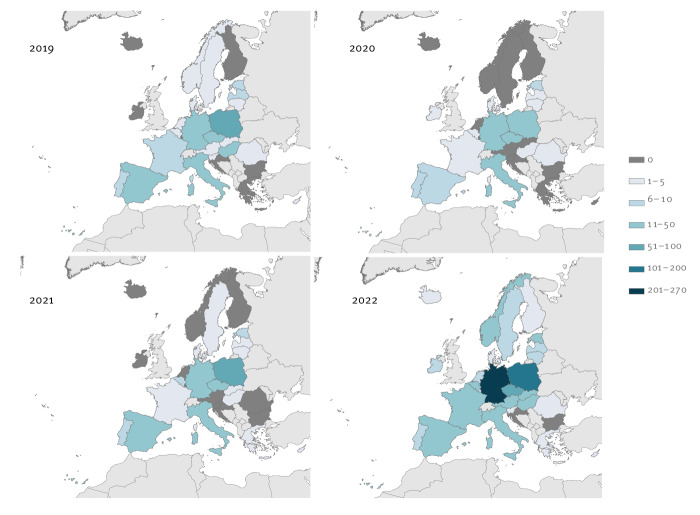
Total number of cases notified with tuberculosis (all forms) reported in Ukrainians, European Union and European Economic Area, 2019–2022 (n = 1,382)

Of all 30 EU/EEA countries included in the study, 27 reported at least one case of TB in a person of Ukrainian origin over the 4-year period of the study, adding up to a total of 1,382 cases. Of these, 780 were reported in 2022, with three countries – Czechia, Germany and Poland – accounting for 71.3% (n = 556) of the notified cases in Ukrainians in the EU/EEA during that year. From 2019 to 2021, these three countries combined reported 56.6% (n = 133) of cases in 2019, 68.9% (n = 113) of cases in 2020 and 64.0% (n = 130) of cases in 2021 (Figure 1).

Ukrainians are defined as individuals holding Ukrainian citizenship or born in Ukraine.

The figure was created with the ECDC Map Maker tool (EMMa).

Countries displayed in light grey are not member states of the European Union and European Economic Area as of January 2024.

**Figure 2:**
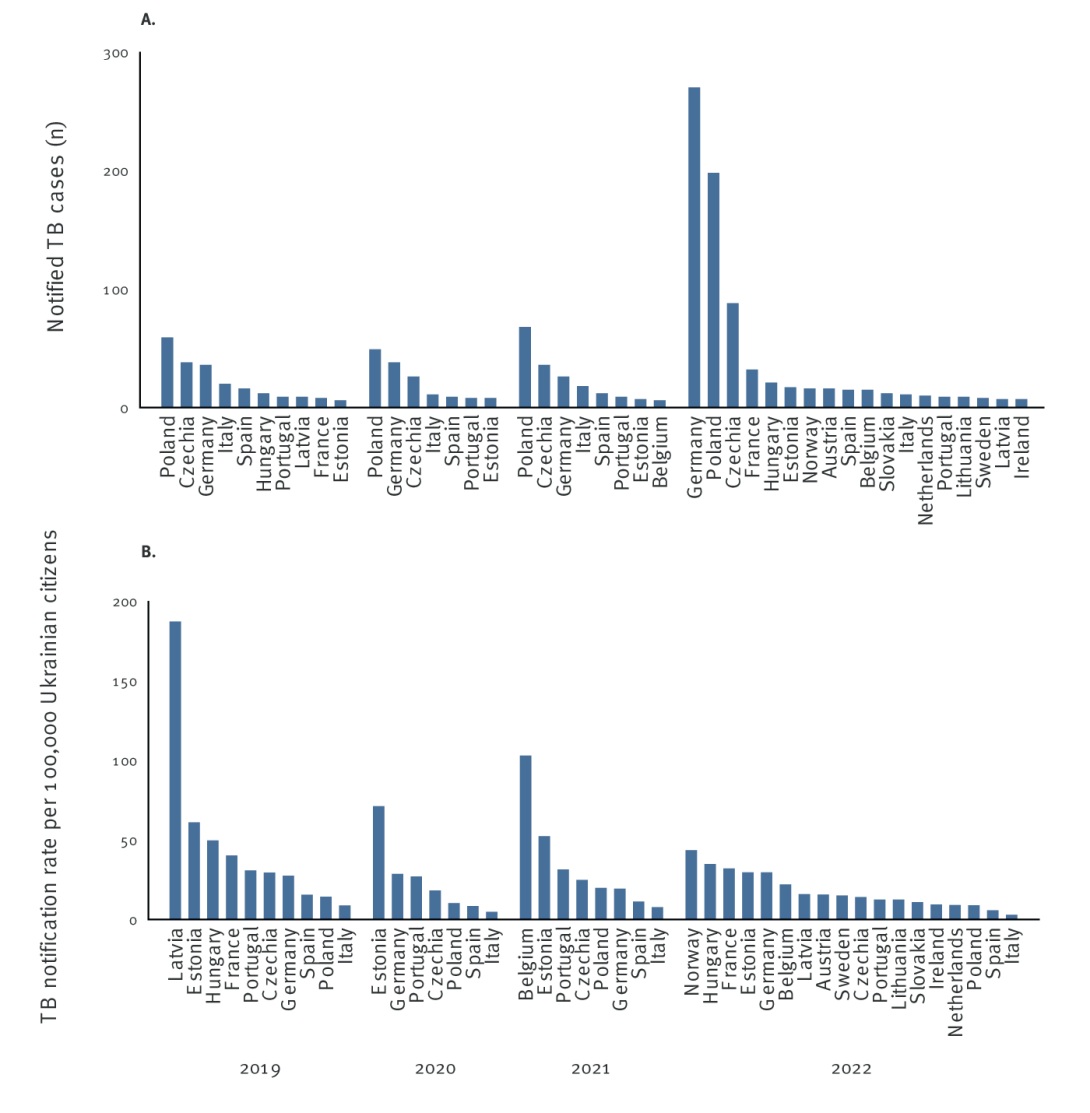
A) Number of notified tuberculosis cases (all forms) in Ukrainians reported in the EU/EEA^a^ (n = 1,382) and B) calculated notification rates per 100,000 Ukrainian citizens in the EU/EEA^b^, 2019–2022

^a^ 2019: 235; 2020: 164; 2021: 203; 2022: 780.

^b^ 2019: 19.6; 2020: 12.6; 2021: 17.0, 2022: 13.4.

The total number of notified TB cases in Ukrainians in the EU/EEA was similar in 2019–2021 (2019: 235; 2020: 164; 2021: 203). However, the number of cases increased almost fourfold in 2022 (n = 780). Cases in Ukrainians accounted for 0.51%, 0.48%, and 0.60% of all TB cases notified in the EU/EEA in 2019, 2020, and 2021, respectively. In 2022, this proportion increased to 2.2%. Country-specific TB notification rates per 100,000 Ukrainian citizens remained below the Ukraine rate (45.5/100,000 in 2021) [1,4] throughout the period of interest, with a few exceptions (Figure 2). At EU/EEA level, the notification rate in Ukrainians decreased from 2019 to 2020 (19.6 to 12.6/100,000 Ukrainian citizens). In 2021, the rate increased to 17.0 per 100,000 and dropped again to 13.4 per 100,000 in 2022 (Table).

Individuals born in Ukraine but holding a different citizenship are not reflected in the denominator.

Countries and time points with less than five cases per year are excluded.

Population data [7,8].

**Table:** Tuberculosis case notifications in Ukrainians in the EU/EEA, 2019–2022

EU/EEA total	2019^a^		2020^a^		2021		2022	
Ukrainian citizens EU/EEA^b^	1,199,185		1,306,104		1,192,058		5,818,113	
- Male-to-female ratio among population	0.9		0.9		0.8		0.6	
Notification rate per 100,000 Ukrainian citizens in the EU/EEA total (95% CI)	19.6	17.2–22.3	12.6	10.8–14.6	17.0	14.8–19.5	13.4	12.5–14.4
TB cases notified in individuals of Ukrainian origin (all forms), n	235		164		203		780	
- Male-to-female ratio among cases	2.0		1.5		2.5		1.4	
Pulmonary TB, n (% of all cases)	213	90.6	151	92.1	184	90.6	709	90.9
Laboratory confirmed^c^, n (% of all cases)	185	78.7	133	81.1	169	83.3	608	77.9
RR/MDR TB^d^, n (% of all^c^)	39	21.1	22	16.5	32	18.9	152	25.0
pre-XDR^e^, n (% of all^c^)	4	2.2	5	3.8	9	5.3	38	6.3
XDR TB^e^, n (% of all^c^)	0	0	0	0	0	0	4	0.7
HIV status available, n (% of all TB cases)	71	30.2	44	26.8	66	32.5	159	20.4
HIV co-infection, n (% of all HIV-positive in cases with an available HIV status)	9	12.7	8	18.2	7	10.6	21	13.2

CI: confidence interval; EEA: European Economic Area; EU: European Union; MDR: multidrug-resistant; pre-XDR: pre-extensively drug-resistant; RR: rifampicin-resistant; TB: tuberculosis; XDR: extensively drug-resistant.

^a^ Including cases for Latvia 2019 and 2020 obtained from the Latvian Public Health Institute.

^b^ Population data [7,8]. Information on sex not provided for Malta and Ireland.

^c^ Laboratory confirmation according to EU case definitions [6].

^d^ RR or MDR TB cases excluding those meeting the pre-XDR or (XDR) TB criteria.

^e^ Pre-XDR/XDR TB cases according to the 2021 World Health Organization definitions [19].

Ukrainians are defined as individuals holding Ukrainian citizenship or born in Ukraine, unless specified otherwise.

## Drug-resistant tuberculosis and tuberculosis/HIV co-infection

**Figure 3:**
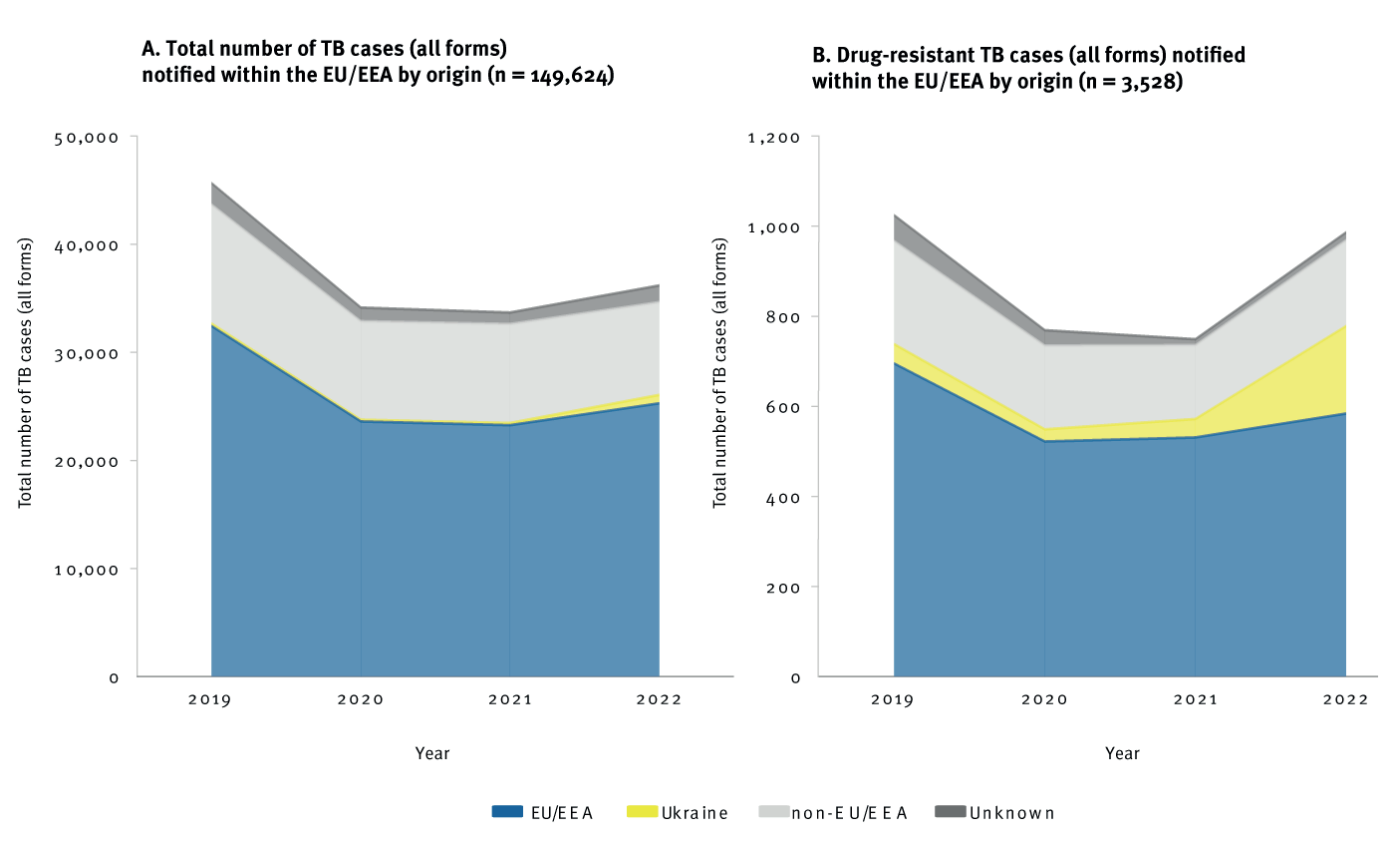
Distribution of A) total number of tuberculosis cases^a^ (n= 149,and B) drug-resistant tuberculosis cases^b^ by origin, notified within the EU/EEA, 2019–2022

EEA: European Economic Area; EU: European Union; TB: tuberculosis.

Among Ukrainians residing in the EU/EEA, the proportion of drug-resistant (DR) TB cases (i.e. resistant to any TB drug) to all laboratory-confirmed cases varied slightly between 2019 and 2021 (2019: 23.2%, n = 43; 2020: 20.3%, n = 27; 2021: 24.3%, n = 41) before rising to 31.9% (n = 194) in 2022. Four cases with extensively drug-resistant (XDR) TB were reported in patients of Ukrainian origin residing in the EU/EEA in 2022; no XDR TB cases were reported in this group in 2019–2021. Similarly, the proportion of DR TB cases reported in the EU/EEA among Ukrainians in comparison to individuals of other origin rose to 19.7% in 2022 compared with previous years (2019: 4.2%, n = 43; 2020: 3.5%, n = 27; 2021: 5.5%, n = 41 in 2021) (Figure 3).

Non-EU/EEA cases here exclude cases of Ukrainian origin. The figure was created with the Canva graphic design tool.

^a^ 2019: 45,634; 2020: 34,135; 2021: 33,676; 2022: 36,179.

^b^ 2019: 1,024; 2020: 769; 2021: 749; 2022: 986.

The figure includes data from Latvia 2019, 2020 while Italy did not report drug-resistant TB status by country-specific origin.

The proportion of Ukrainian TB cases with a concomitant HIV infection in the EU/EEA was consistently lower than the level of 23.0%, 21.9%, 20.3% and 18% of the cases with documented HIV status reported in Ukraine between 2019 and 2022 [1,3,4]. However, information regarding the HIV status of cases in Ukrainians in the EU/EEA was available only in 20.4% of all cases in 2022 of which 13.2% were HIV-positive (Table).

## Discussion

In 2022, the absolute number of notified TB cases among Ukrainians in the EU/EEA showed an almost fourfold increase (n = 780) compared with 2019–2021. This can be attributed to the increase in the number of people from Ukraine residing in the EU/EEA after the start of the Russian invasion of Ukraine.

In contrast to the notification rates reported in Ukraine (45.5/100,000 in 2021), average TB notification rates in the EU/EEA remained at low levels in 2022 (13.4/100,000 Ukrainian citizens), consistent with previous years. One possible explanation for this observation is the demographic structure of the migrant population, which differs from the demographics of highly affected population groups in Ukraine regarding sex and age distribution [1,4,7,8]. Based on WHO estimates, under-diagnosis and under-reporting in the host countries can also be assumed [9].

Notification rates were calculated using citizenship-based datasets for the denominator. However, the TB surveillance data included cases involving both Ukrainian citizens and individuals born in Ukraine who may hold a different citizenship. Consequently, there was a mismatch between the numerator and denominator for countries reporting TB cases based on the country of birth [4]. Sensitivity analysis of notification rates using different methods of defining origin (by citizenship or country of birth) showed similar trends on a negative binomial regression for the years 2019–2021. This information can be seen in Supplementary Figure S2.

Our results reflect the high prevalence of DR TB observed in Ukraine [1,3,4]. More than 30% of all laboratory confirmed Ukrainian cases in the EU/EEA were affected by DR TB. This finding highlights the importance of timely case identification, drug susceptibility testing followed by the initiation of appropriate treatment and continuity of care in this population [10].

Recently published data from Czechia and Slovakia [11] indicate that local transmission linked to newly arrived Ukrainians with TB has not been observed in the host countries. However, continued screening efforts among individuals with increased risk for TB [12] and in settings with higher risk for TB transmission [13] should be encouraged. General screening among Ukrainian migrant populations is likely to result in higher case detection rates. Nevertheless, concerns regarding acceptability, feasibility, stigmatisation and hesitancy have been reported in several EU/EEA countries [14-16]. Further, the WHO Regional Office for Europe and ECDC do not recommend TB screening for Ukrainians entering the EU/EEA [12].

Our study has several limitations. Our results are based on routinely collected retrospective data provided by the EU/EEA countries and rely on accurate reporting. Definitions across datasets were partially not uniform, especially regarding the definition of origin. Further, over- or underestimation of notification rates is possible due to fluctuations of the population size.

## Conclusion

Migrant-sensitive healthcare services for Ukrainians, such as ambulatory care models, socioeconomic support and language-appropriate counselling can encourage early presentation and ensure retention in treatment, thereby limiting chances of transmission [17,18]. The utilisation of molecular epidemiology can aid contact tracing efforts and allow the identification of transmission chains. Strengthening of the EU/EEA-wide surveillance network with upscaling of conventional and molecular epidemiology capacities and uniform reporting strategies would help improve cross-border TB control in the area.

## Supplementary Data

1463000 Supplementary Material1

## Supplementary Data

3199000 Supplementary Material2
