# Supplementary material for: Tuberculosis in people of Ukrainian origin in the European Union and the European Economic Area, 2019 to 2022
Source: Euro Surveill. 2024 Mar 21;29(12):2400094. doi: 10.2807/1560-7917.ES.2024.29.12.2400094 (PMC11063674; doi:10.2807/1560-7917.ES.2024.29.12.2400094)
Supplement: Supplementary Material2 [file 24-00094_STOYCHEVA_Supplementary_Figure2.pdf]

Supplementary Figure S2. Predictions for country-specific notification rates in a population defined by country of birth (COB) versus a population defined by citizenship (CTZ) in countries and timepoints with datasets available by both COB & CTZ.

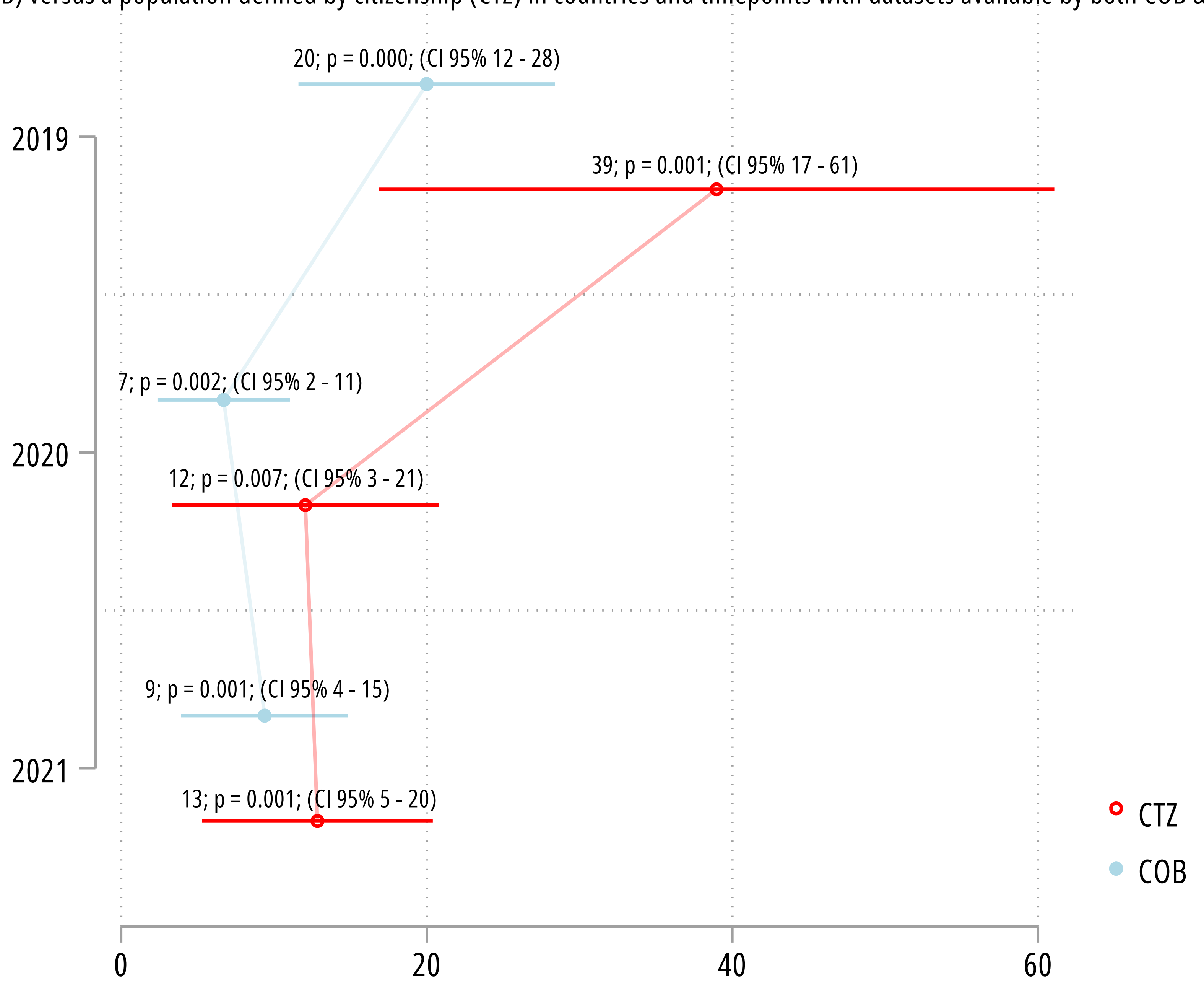

This supplementary material is hosted by *Eurosurveillance* as supporting information alongside the article 'Tuberculosis in people of Ukrainian origin in the European Union and the European Economic Area, 2019 to 2022', on behalf of the authors, who remain responsible for the accuracy and appropriateness of the content. The same standards for ethics, copyright, attributions and permissions as for the article apply. Supplements are not edited by *Eurosurveillance* and the journal is not responsible for the maintenance of any links or email addresses provided therein.
